# Predictors of lack of response to methotrexate in juvenile idiopathic arthritis associated uveitis

**DOI:** 10.1093/rheumatology/keae079

**Published:** 2024-02-08

**Authors:** Chiara Mapelli, Elisabetta Miserocchi, Marco Nassisi, Gisella B Beretta, Luca Marelli, Gaia Leone, Achille Marino, Cecilia Chighizola, Gilberto Cincinelli, Teresa Giani, Paolo Nucci, Francesco Viola, Giovanni Filocamo, Francesca Minoia, Pediatric Rheumatology Associated Group of the Milan Area, Carlo Agostoni, Francesco Baldo, Lucia Baselli, Stefania Costi, Fabiana Di Stasio, Maurizio Gattinara, Stefano Lanni, Antonella Petaccia, Martina Rossano, Federica Vianello

**Affiliations:** Ophthalmology Unit, Fondazione IRCCS Ca’ Granda Ospedale Maggiore Policlinico, Milan, Italy; San Raffaele Scientific Institute, Milan, Italy; Ophthalmology Unit, Fondazione IRCCS Ca’ Granda Ospedale Maggiore Policlinico, Milan, Italy; University of Milan, Milan, Italy; University of Milan, Milan, Italy; Pediatric Immuno-Rheumatology Unit, Fondazione IRCCS Ca’ Granda Ospedale Maggiore Policlinico, Milan, Italy; University of Milan, Milan, Italy; Ophthalmology Unit, Fondazione IRCCS Ca’ Granda Ospedale Maggiore Policlinico, Milan, Italy; Pediatric Rheumatology Unit, ASST Gaetano Pini, Milan, Italy; University of Milan, Milan, Italy; Pediatric Rheumatology Unit, ASST Gaetano Pini, Milan, Italy; University of Milan, Milan, Italy; Pediatric Rheumatology Unit, ASST Gaetano Pini, Milan, Italy; Department of Pediatrics, AOU Meyer, Florence, Italy; University of Milan, Milan, Italy; Ophthalmology Unit, San Giuseppe Hospital, IRCCS Multimedica, Milan, Italy; Ophthalmology Unit, Fondazione IRCCS Ca’ Granda Ospedale Maggiore Policlinico, Milan, Italy; University of Milan, Milan, Italy; Pediatric Immuno-Rheumatology Unit, Fondazione IRCCS Ca’ Granda Ospedale Maggiore Policlinico, Milan, Italy; Pediatric Immuno-Rheumatology Unit, Fondazione IRCCS Ca’ Granda Ospedale Maggiore Policlinico, Milan, Italy

**Keywords:** uveitis, juvenile idiopathic arthritis, methotrexate, biologics

## Abstract

**Objectives:**

To investigate clinical features associated with lack of response to MTX in juvenile idiopathic arthritis associated uveitis (JIA-U).

**Methods:**

Clinical records of JIA-U patients were retrospectively reviewed. Differences among variables were assessed by Mann–Whitney and χ^2^ or Fisher’s exact tests as appropriate. Association between predictors and requirement of a biological disease-modifying antirheumatic drug (bDMARD) was evaluated by univariate Cox regression analysis and Kaplan–Meier curves. A multivariable logistic model was applied to estimate strength of association, adjusting for potential confounders.

**Results:**

Data from 99 JIA-U patients treated with MTX were analysed (82.8% female), with a mean follow up of 9.2 years and a mean age at uveitis onset of 5.7 years. In 65 patients (65.7%) at least one bDMARD to control uveitis was required. Children requiring a bDMARD for uveitis had lower age at JIA and uveitis onset, more frequent polyarticular course, higher frequency of bilateral uveitis at onset and higher prevalence of systemic steroids’ use. Despite similar frequency of ocular damage at onset, MTX non-responders showed a higher percentage of ocular damage at last visit. Younger age at JIA onset, polyarticular course and a history of systemic steroids’ use resulted independent factors associated to lack of response to MTX at Cox regression analysis. Kaplan–Meier curves and the multivariate model confirm the independent role of both polyarticular course and systemic steroids’ use.

**Conclusions:**

Younger age at JIA onset, polyarticular course and a history of systemic steroids’ use are predictors of a worse response to MTX in JIA-U.

Rheumatology key messages

- No features were recognized as predictive factors for treatment response in juvenile idiopathic arthritis associated uveitis (JIA-U) so far.
- Refractory course in uveitis is associated to a higher incidence of ocular damage.
- Early JIA onset, polyarthritis and systemic steroids’ use predict worse response to MTX in JIA-U.

## Introduction

Uveitis is the main extraarticular complication of juvenile idiopathic arthritis (JIA), affecting 10–20% of children with JIA [1, 2]. JIA-associated uveitis (JIA-U) mainly involves the anterior chamber of the eye, its onset is usually insidious and mostly asymptomatic. Optimized screening protocols and the introduction of disease-modifying antirheumatic drugs (DMARDs) earlier in the disease course significantly improved the outcome of JIA-U patients [3–6]. Nevertheless, JIA-U is still a notable cause of visual morbidity, with damage still reported in about half of patients and eye surgery often required [2, 7–9].

The presence of antinuclear antibodies (ANA), oligoarticular course, early onset of arthritis and female gender have been associated with a higher risk of developing uveitis in children with JIA [7, 10, 11]. Risk factors for poor visual prognosis in JIA-U patients include the activity grade of uveitis at onset, the presence of ocular damage at the first ophthalmologic evaluation, uveitis antedating arthritis and male gender [6, 12–16]. However, no clinical features have been widely recognized as predictive factors for lack of response to treatment in JIA-U, so far. With this background, the principal aim of our study was to investigate the clinical features potentially associated with an inadequate response to methotrexate (MTX) in a long-term cohort of patients with JIA-U.

## Methods

### Study population

Clinical records of patients with JIA-U treated at the two tertiary Pediatric Rheumatology Units in Milan, Italy, between 2000 and 2020 were retrospectively reviewed. All patients received a diagnosis of JIA according to the International League Against Rheumatism (ILAR) classification criteria [17] and chronic anterior uveitis according to the Standardization Uveitis Nomenclature (SUN) Working Group criteria [18]. To be included in the study, patients should have been treated with MTX and a follow-up of at least 6 months should be available.

Data were anonymously collected in an electronic database from standardized forms, in which demographic data and clinical features regarding both JIA and uveitis were collected, together with therapeutic choices and outcome. Routinely performed ophthalmological visits included evaluation of best-corrected visual acuity (BCVA), anterior chamber (AC) slit lamp examination with activity grade according to SUN criteria, intraocular pressure (IOP) assessment and ophthalmoscopy. The presence of ocular damage at baseline and at last visit was also reported and included band-keratopathy, posterior synechiae, cataract, chronic macular changes, glaucoma, optic disc atrophic changes, phtisis bulbi and visual loss <4/10. Inactive uveitis was defined according to the Multinational Interdisciplinary Working Group for Uveitis in Childhood (MIWGUC) as slit lamp AC grade <0.5+, absence of optic disc or macular oedema and absence of vitreous haze (<0.5+) [19].

To evaluate features potentially associated to a lack of response to MTX, the population was divided into two groups: patients who responded to MTX and patients who required at least one biological DMARD (bDMARD) to control uveitis. Adequately controlled uveitis was defined as slit lamp AC grade <0.5+, requiring <2 drops/day of topical steroids, without systemic steroids and with no new ocular complications for at least 3 months [20, 21]. As clinical practice, both centres started bDMARD in addition to MTX, except when an intolerance to MTX was reported, according to international recommendations [20, 21]. In order to reduce a potential bias due to clinical indication, patients who required the introduction of a bDMARD only for active arthritis were excluded.

The present study was conducted following the principles of the Declaration of Helsinki and the study protocol was approved by the local Institutional Review Board (Comitato Etico Territoriale Lombardia 3; Local EC number approval 1256).

### Statistical analysis

Demographic and clinical characteristics were reported as median and interquartile range (IQR) or mean ± standard deviation for continuous variables and percentages for categorical variables, as appropriate. Differences in continuous variables between the two groups were assessed by performing Mann–Whitney non-parametric tests. Association between categorical variables was tested by using χ^2^ or Fisher’s exact tests as appropriate.

A univariate Cox regression analysis was applied in order to estimate the strength of association between predictors and lack of response. The primary outcome measure was the introduction of at least one bDMARD to control uveitis. The time to event considered in the Cox regression analysis was the duration between the onset of uveitis and the introduction of bDMARD. Patients who did not receive a bDMARD were censored at the last ophthalmology visit available. A multivariable model was then estimated using predictors that had a *P*-value <0.1 in order to account for potential confounders. Finally, the occurrence of bDMARD introduction was analysed using the Kaplan–Meier method, and time course differences among subgroups were compared using the log-rank test. IBM Statistical Package for the Social Sciences (SPSS) software (v. 21.0, IBM, Chicago, IL, USA) was used.

## Results

A total of 99 patients with JIA-U and treated with MTX were included in the study, with a large female predominance (82.8%). The mean age at uveitis onset was 5.7 ± 3.8 years, and the mean follow-up was 9.2 ± 4.7 years. Almost all patients developed uveitis after JIA onset (96.9%), with a mean interval time of 1.86 ± 3.13 years. Table 1 summarizes the main clinical and ophthalmological features observed in the cohort.

**Table 1.** Demographic, clinical and ophthalmological features of the 99 patients with JIA-U collected

	Patients with JIA-U N 99
Males, *n* (%)	17 (17.2)
Age at uveitis onset (yr), mean ± SD	5.7 ± 3.8
Age at JIA onset (yr), mean ± SD	3.9 ± 3.1
Follow-up (yr), mean ± SD	9.2 ± 4.7
Time between JIA and uveitis onset, mean ± SD	1.9 ± 3.1
JIA features	
Polyarticular course, *n* (%)	25 (25.3)
Active joint count at JIA onset, median (IQR)^a^	1.0 (2.0)
Active arthritis at uveitis onset, *n* (%)	54/86 (62.8)
On MTX treatment at uveitis onset, *n* (%)	63 (63.6)
Ophthalmological features at uveitis onset	
Bilateral involvement	64 (64.7)
Best Corrected Visual Acuity^c^, mean (SD), LogMAR	0.04 (0.11)
SUN activity grade in AC, median (IQR)^b^	2.0 (1.0)
Ocular damage	20/87 (23.0)
Laboratory markers	
ANA positivity	91 (91.9)
Positive acute phase reactants at uveitis onset^d^	34/55 (61.8)
History of systemic steroids’ use, *n* (%)	38 (38.4)

Data were calculated on the overall population, unless otherwise specified.

a Data available on 88 subjects.

b Data available on 71 subjects.

c Data available on 56 subjects and calculated in the worse eye.

d Positive acute phase reactants includes C-reactive protein and/or erythrocyte sedimentation rate. AC: anterior chamber; ANA: antinuclear antibodies; IQR: interquartile range; JIA: juvenile idiopathic arthritis; JIA-U: JIA-associated uveitis; SUN: standardized uveitis nomenclature.

**Figure 1.**
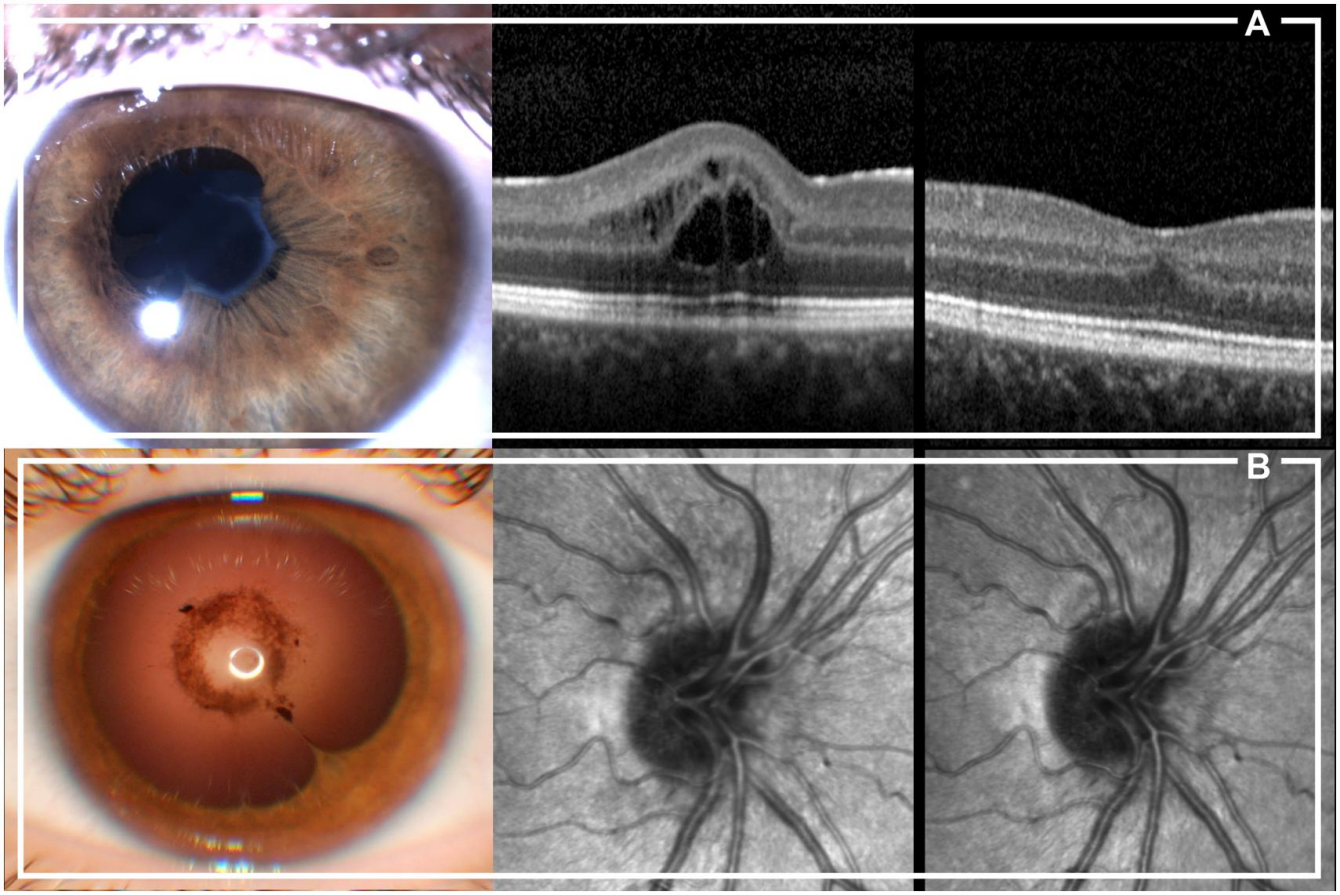
Ocular complications in patients with JIA not responsive to methotrexate treatment. (**A**) Anterior segment image of a 14-year-old female patient with posterior synechiae causing an irregularity of the pupil shape (left); an optical coherence tomography imaging of the macula shows cystoid edema (middle). While posterior synechiae remained stable over the years, cystoid edema resolved after introducing a biologic disease-modifying antirheumatic drug (bDMARD) (right). (**B**) A 12-year-old female patient showing posterior synechiae at the slit lamp examination image (left), and the presence of optic disc swelling on infrared fundus image (middle). The introduction of bDMARD resolved the optic disc swelling after three months (right)

In 34 patients a therapy with MTX was effective to achieve ocular clinical remission, while in 65 patients (65.7%) at least one bDMARD to control uveitis was required, after a median time of 3.3 years. Fifty-five patients (84.6%) started the bDMARD in addition to MTX, while in 10 cases MTX was stopped due to intolerance. Sixty-three patients (63.6%) were MTX-naive at uveitis onset (20 patients—58.8% in the MTX-responders group and 43 patients—66.2% in the bDMARD group, without significant difference, *P* = 0.514). As shown in Table 2, children requiring a bDMARD had a lower age at JIA and uveitis onset, a more frequent polyarticular course, a higher frequency of bilateral uveitis at onset and a higher prevalence of a history of systemic steroids’ use. No difference was observed in uveitis activity grade at onset. Despite similar frequency of ocular damage at diagnosis, patients not responsive to MTX showed a higher percentage of ocular damage (Fig. 1) at last visit (61.5% *vs* 32.4% *P* = 0.007). Nevertheless, the final visual acuity in the worse eye was not statistically different between the two groups (0.01 ± 0.03 LogMAR in MTX patients and 0.06 ± 0.21 LogMAR in bDMARD patients; *P* = 0.317). Children treated with a bDMARD for uveitis were more frequently males, although the difference did not reach statistical significance (*P*-value = 0.111), probably due to the limited absolute number of male patients in our cohort.

**Table 2.** Demographic, clinical and ophthalmological features of the 99 patients with JIA-U, stratified according to response to treatment

	JIA-U patients responsive to MTX N 34	JIA-U patients requiring bDMARD to control uveitis N 65	*P*-value
Males, n (%)	3 (8.8)	14 (21.5)	0.111^b^
Age at JIA onset (yr), mean (SD)	4.5 (3.7)	3.3 (2.7)	0.022^c^
Age at uveitis onset (yr), mean (SD)	6.8 (3.9)	5.4 (3.7)	0.014^c^
Follow-up (yr), mean (SD)	8.4 (4.6)	9.6 (4.7)	0.265^c^
Time between JIA and uveitis onset, mean ± SD	1.9 (3.2)	1.7 (3.1)	0.991^c^
JIA features			
Polyarticular course, *n* (%)	5 (14.7)	22 (33.8)	0.010^b^
Active joint count at JIA onset, median (IQR)	1.0 (2.0)	1.5 (3.0)	0.950^b^
Active arthritis at uveitis onset	17/30 (57.6)	37/56 (66.1)	0.390^b^
On MTX treatment at uveitis onset, *n* (%)	20 (58.8)	43 (66.2)	0.514^b^
History of systemic steroids’ use, *n* (%)	4 (11.76)	34 (52.3)	<0.001^b^
Ophthalmological features at uveitis onset			
Bilateral involvement	17 (50.0)	47 (72.3)	0.024^b^
Best Corrected Visual Acuity,^e^ mean (SD), LogMAR	0.05 (0.15)	0.04 (0.10)	0.801^c^
SUN activity grade in AC, median (IQR)^a^	2.0 (1.4)	1.0 (1.0)	0.557^f^
Ocular damage	4/30 (13.3)	16/57 (28.1)	0.180^b^
Laboratory markers			
ANA positivity	31 (91.2)	60 (92.3)	1.000^b^
Positive acute phase reactants at uveitis onset^d^	12/22 (54.5)	22/33 (66.7)	0.407^b^

Data were calculated on the overall population, unless otherwise specified.

a Data available on 71 subjects.

b Fisher’s exact test.

c Mann–Whitney *U* test.

d Positive acute phase reactants includes C-reactive protein and/or erythrocyte sedimentation rate.

e Data available on 56 subjects and calculated in the worse eye.

f χ^2^ test.

AC: anterior chamber; ANA: antinuclear antibodies; cDMARD: conventional DMARD; IQR: interquartile range; JIA: juvenile idiopathic arthritis; JIA-U: JIA-associated uveitis; LogMAR: logarithm of the minimum angle of resolution; MTX: methotrexate; SUN: standardized uveitis nomenclature.

**Figure 2.**
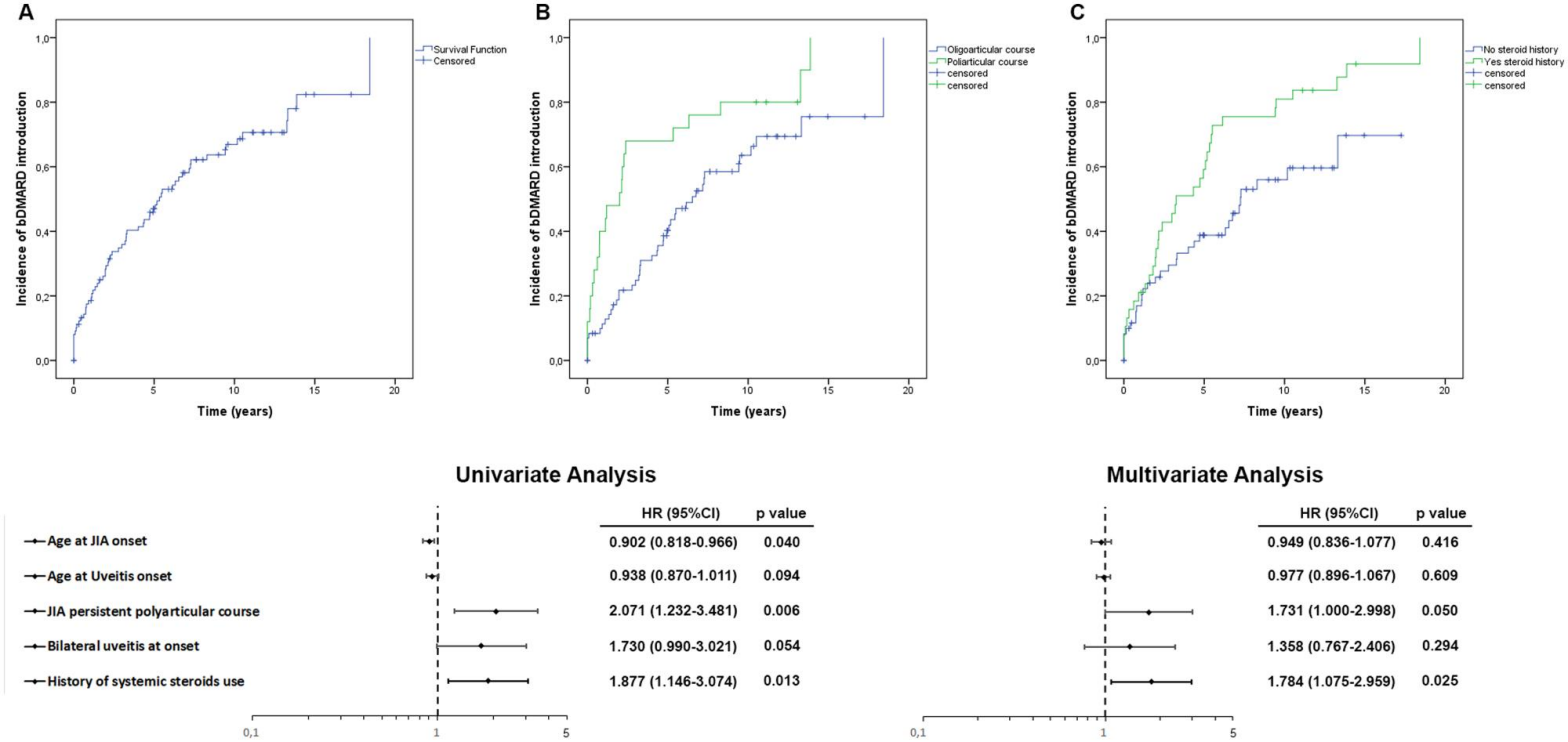
Kaplan–Meier cumulative incidence analysis and Cox regression analysis of the introduction of bDMARD in patients with JIA. (**A**) Sixty-five patients required a bDMARD for uveitis by the end of follow-up period (1.2 per 10 person-years). (**B**, **C**) Cumulative incidence rates differed between patients with polyarthritis and oligoarthritis (B, log-rank test: *P* = 0.005), and between patients with and without a history of systemic steroids’ use (C, log-rank test: *P*=0.011). The forest plot of hazard ratios (bottom) includes results from univariate (on the left) and multivariate (on the right) analyses and encompasses all risk factors with P-values less than 0.1 by Cox regression analysis. Both polyarticular course and history of systemic steroids’ use correlated with a higher risk of requiring a bDMARD in JIA-U

Cox regression analysis showed that a younger age at JIA onset, polyarticular course and a history of systemic steroids’ use were independent factors associated to a lack of response to MTX (Fig. 2, Table 3). To note, 75% of patients requiring a bDMARD were younger than 5 years old at JIA onset and younger than 12 years old when the first bDMARD for uveitis was started. In the multivariate model, only polyarticular course (hazard ratio [HR]: 1.731 [1.000–2.998, 95% confidence interval], *P* = 0.05) and a history of systemic steroids’ use (HR: 1.784 [1.075–2.959], *P* = 0.025) remained statistically significant (Table 3). Kaplan–Meier curves analysing the introduction of bDMARDs to control uveitis in the cohort are shown in Fig. 2. In the overall cohort, the median survival time was 5.3 years with an incidence rate of 1.2 per 10 person-years (Fig. 2A). Cumulative incidence rates differed notably between patients with a polyarticular course and those with a persistent oligoarticular course (median survival time 2 years; incidence rate 2.2 per 10 person-years *vs* median survival time 6.5 years; incidence rate 1 per 10 person-years, respectively; log-rank test: *P* = 0.005) (Fig. 2B). Furthermore, patients with a history of systemic steroids’ use had a shorter median survival time (3.3 years) and a higher incidence rate (1.8 per 10 person-years) compared with patients without this history (median survival time 7.3 years; incidence rate 0.9 per 10 person-years; log-rank test: *P* = 0.011) (Fig. 2C).

**Table 3.** Cox regression analysis assessing putative predictors of lack of response of JIA-U to MTX

	Univariate analysis		Multivariate analysis	
	HAZARD RATIO (95% CI)	*P* value	HAZARD RATIO (95% CI)	*P* value
Age at JIA onset	0.902 (0.818–0.966)	0.040	0.949 (0.836–1.077)	0.416
Age at uveitis onset	0.938 (0.870–1.011)	0.094	0.977 (0.896–1.067)	0.609
Male gender	1.535 (0.833–2.831)	0.170	—	—
JIA polyarticular course	2.071 (1.232-3.481)	0.006	1.731 (1.000-2.998)	0.050
ANA positivity	1.393 (0.553–3.511)	0.482	—	—
Time between JIA and uveitis onset	1.010 (0.940–1.085)	0.782	—	—
Positive acute phase reactants at uveitis onset^a^	1.371 (0.658–2.855)	0.400	—	—
Active arthritis at uveitis onset	1.246 (0.704–2.205)	0.450	—	—
Bilateral uveitis at onset	1.730 (0.990–3.021)	0.054	1.358 (0.767–2.406)	0.294
Ocular damage at uveitis onset^c^	1.361 (0.752–2.462)	0.309	—	—
SUN activity grade in AC at uveitis onset^b^	0.794 (0.555–1.137)	0.207	—	—
History of systemic steroids’ use	1.877 (1.146–3.074)	0.013	1.784 (1.075–2.959)	0.025

95% CI: 95% confidence interval; AC: anterior chamber; ANA: antinuclear antibodies; JIA: juvenile idiopathic arthritis; SUN: standardized uveitis nomenclature.

a 44 missing data.

b 28 missing data.

c 12 missing data.

## Discussion

Risk factors for uveitis development in JIA and parameters associated with poor outcome in JIA-U have been previously assessed [6, 7, 10–13, 16]. However, to the best of our knowledge, no widely recognized clinical variable has been associated to the lack of response to treatment in patients with JIA-U. To address this point, we evaluated the potential predictors of lack of response to MTX in a long-term cohort of patients with JIA-U, followed up in two tertiary centres.

The demographic and clinical features observed in our population were similar to larger cohorts in literature, with a striking prevalence of females, ANA positivity and early onset of JIA. Interestingly, 62% of patients in which this data was available presented an elevation of acute phase reactants at the time of uveitis diagnosis, supporting the previously suggested role of erythrocyte sedimentation rate (ESR) as a marker for uveitis occurrence in JIA [22–25]. The presence of ocular damage at the first ophthalmological evaluation in 23% of JIA-U patients, even if slightly below that reported in previous studies [13, 26, 27], highlights the importance of accurate screening protocols in JIA patients.

MTX is widely agreed to be the first-line systemic treatment in JIA-U. SHARE recommendations for JIA-U strongly suggest an early introduction of MTX in patients with poor prognostic factors and in all JIA-U patients who do not reach a stable uveitis’ inactivity state within 3 months [28]; likewise, the Childhood Arthritis and Rheumatology Research Alliance Consensus (CARRA) Treatment Plans includes MTX for all JIA-U children with a persistent, progressive or recurrent uveitis despite topical treatment [20]. MTX was observed to reduce the risk of visual loss [3, 6] and to prevent uveitis onset in JIA patients when started early after articular onset [4, 5]. However, in a recent study by Tirelli *et al.* MTX, despite its high effectiveness in the early stages, was associated with poor JIA-U control in the long-term, especially in children already on MTX treatment at the onset of uveitis [27]. Moreover, in the ICON-JIA cohort, MTX was not a predictor for achieving uveitis’ inactivity nor reduction of relapses or better 2-year outcome [26]. In our cohort, similar to Tirelli *et al.* data, more than half of children required at least one biological DMARD to control uveitis. Notably, we did not find any significant difference in MTX response between patients MTX-naive or already on MTX at uveitis onset.

Considering the key role of uncontrolled inflammation in the outcome of JIA-U [29], early identification of patients at high risk of an incomplete response to first-line treatment is crucial. Herein, we identified early age at JIA onset, polyarticular course and a history of systemic steroids’ use as three independent factors associated to a lack of response to MTX.

Early onset of arthritis is a widely recognized risk factor for uveitis in patients with JIA [7, 10, 11] and interestingly, in the ICON-JIA cohort, younger age at JIA onset (<4 years old) was also associated with a higher risk of not reaching an inactivity state of uveitis at 6 months [26]. Oligoarticular course has been associated with a higher risk of JIA-U in several studies [3, 7–9, 11, 30]; however, no specific JIA subtype has ever been associated with poor outcome in JIA-U so far. In our cohort, even after excluding JIA patients with exclusive indication to bDMARDs for active arthritis, the polyarticular course resulted in a significant and independent factor associated to an inadequate control of uveitis with MTX. Of note, Giancane *et al.* compared outcomes between the MTX-era and the biologic-era in a retrospective cohort of patients with JIA: in the polyarthritis group, prevalence of uveitis did not show any significant improvement since biologic introduction [31]. Together with our data, this can suggest that JIA patients with polyarticular course, once they developed uveitis, might be at higher risk of a more aggressive form.

A history of systemic steroids’ use resulted in the risk factor most strongly associated to the lack of response to MTX. Unfortunately, steroid cumulative dose could not be included in the analysis, due to several missing data. Patients who received systemic steroids had a date of uveitis onset equally distributed over the study time. Although we do not collect the data on arthritis activity specifically at the time of steroids treatment, all patients had uncontrolled uveitis and they did not differ from the overall population in terms of polyarthritis’ frequency. All together these data suggest that systemic steroids may have been used more frequently in patients with a more aggressive ocular disease, supporting an early introduction of a bDMARD in patients with an inadequate uveitis’ control.

Male gender has been variably associated to poor visual outcome in several studies [15, 16, 21, 32], while others did not confirm these results [33, 34]. In our cohort, a bDMARD was required by a higher percentage of males, but values did not reach statistical significance. The high variability of data on male gender in literature could be explained by the small sample size of males in JIA-U cohorts, which may limit the accuracy of statistical evaluation. Although the uveitis activity grade in AC has been associated with visual outcome [6, 12, 14, 24], we did not observe a role in predicting lack of response to MTX for AC activity grade, nor for ESR levels at diagnosis, ANA positivity or the presence of ocular damage at first ophthalmological evaluation. Considering damage as the result of acute inflammation, cumulative persistent disease activity and medications’ toxicity, its greater frequency in patients not responsive to MTX may be considered a marker of uncontrolled uveitis and stresses the importance to promptly identify these patients to rapidly start an adequate treatment to prevent long-term complications.

Our study is limited by the retrospective nature of data and the relatively small sample size, which nevertheless is representative of the real-life clinical experience in tertiary centres. Moreover, such observational design has a potential for residual and unmeasured confounding that may not have been adequately considered. Furthermore, treatment strategies were based on caring physicians’ choices and were not standardized; however, all patients were treated in two tertiary referral centres, in which international agreed guidelines are applied and local protocols shared.

In conclusion, younger age at JIA onset, polyarticular course and a history of systemic steroids’ use are predictors of a worse response to MTX in JIA-U. Children resistant to first-line systemic treatment need a prompt recognition and additional strategies to improve long-term outcome.

## Supplementary Material

keae079_Supplementary_Data

## Data Availability

All data relevant to the study are included in the article. Data are available upon request from Dr Francesca Minoia (francesca.minoia@policlinico.mi.it).
