# Supplementary material for: Predictors of lack of response to methotrexate in juvenile idiopathic arthritis associated uveitis
Source: Rheumatology (Oxford). 2024 Feb 8;64(2):798–804. doi: 10.1093/rheumatology/keae079 (PMC11781584; doi:10.1093/rheumatology/keae079)
Supplement: keae079_Supplementary_Data [file keae079_supplementary_data.docx]

**Mapelli C et al, *Predictors of lack of response to methotrexate in juvenile idiopathic arthritis associated uveitis***

**Supplementary Data S1. – List of members of the Pediatric Rheumatology Associated Group of the Milan Area**

Carlo Agostoni, MD PhD (Fondazione IRCCS Ca’ Granda Ospedale Maggiore Policlinico, Milan, Italy), Francesco Baldo (ASST Gaetano Pini, Milan, Italy), Lucia Baselli, MD (Fondazione IRCCS Ca’ Granda Ospedale Maggiore Policlinico, Milan, Italy), Stefania Costi (ASST Gaetano Pini, Milan, Italy), Fabiana Di Stasio (Fondazione IRCCS Ca’ Granda Ospedale Maggiore Policlinico, Milan, Italy), Maurizio Gattinara (ASST Gaetano Pini, Milan, Italy), Stefano Lanni, MD PhD (Fondazione IRCCS Ca’ Granda Ospedale Maggiore Policlinico, Milan, Italy), Antonella Petaccia, MD ((Fondazione IRCCS Ca’ Granda Ospedale Maggiore Policlinico, Milan, Italy), Martina Rossano, MD (Fondazione IRCCS Ca’ Granda Ospedale Maggiore Policlinico, Milan, Italy), Federica Vianello (Fondazione IRCCS Ca’ Granda Ospedale Maggiore Policlinico, Milan, Italy)
